# Comparison of treatment effects between two types of facemasks in early Class III patients

**DOI:** 10.1002/cre2.694

**Published:** 2022-12-05

**Authors:** Nam‐Ki Lee, So‐Hyun Kim, Jae Hyun Park, Dong‐Whan Son, Tae‐Hyun Choi

**Affiliations:** ^1^ Department of Orthodontics Seoul National University Bundang Hospital Seongnam South Korea; ^2^ Postgraduate Orthodontic Program, Arizona School of Dentistry & Oral Health A.T. Still University Mesa Ariz USA; ^3^ Graduate School of Dentistry Kyung Hee University Seoul South Korea; ^4^ Department of Prosthodontics Seoul National University Bundang Hospital Seongnam South Korea

**Keywords:** cephalometric comparison, Class III malocclusion, orthopedic treatment, treatment effects

## Abstract

**Objective:**

To compare the short‐term treatment effects between two types of facemasks in skeletal Class III patients.

**Materials and Methods:**

This retrospective study included 40 skeletal Class III subjects (mean age: 7.7 years) who had been treated with protraction facemasks with forehead straps (PFFS) or Petit type facemasks (PTF). Lateral cephalograms were analyzed at pretreatment (T1) and posttreatment (T2) with an average interval of 9 months.

**Results:**

At T1, PFFS and PTF groups showed similar sagittal, vertical dentoskeletal patterns. From T1 to T2, both groups presented significant forward movement of the maxilla, posterior movement and clockwise rotation of the mandible (all *p* < .001), labioversion of the maxillary incisors, and linguoversion of the mandibular incisors. They showed increased overjet (*p* < .001). Although there were no significant differences in the number of changes in most dentoskeletal variables between the two groups, the PFFS group showed more anterior rotation of the palatal plane and backward rotation of the mandible compared to the PTF group, resulting in a significant decrease (−0.42 mm) in overbite (*p* < .05).

**Conclusions:**

Both PFFS and PTF showed no significant differences in most skeletal and dental changes, except for overbite. These findings might be helpful for clinicians in selecting the types of facemasks for growing Class III malocclusion patients.

## 1 INTRODUCTION

Skeletal class III malocclusion is one of the most challenging problems encountered by orthodontic clinicians because of its heredity and multifactorial influence in etiology (Cruz et al., 2008), the unpredictability of growth (Ngan, 2002), and its complexity in combinations of skeletal and dentoalveolar malocclusions; maxillary retrognathia, mandibular prognathia, or both (Proffit et al., 2012). Ellis and McNamara reported that 62%–67% of skeletal Class III malocclusion is due to maxillary underdevelopment, which can be treated by maxillary protraction with a facemask (Ellis & McNamara, 1984). Many studies to search for golden timing for facemask therapy (Kapust et al., 1998; Saadia & Torres, 2000; Suda et al., 2000) stated that early mixed dentition is a good period for maxillary protraction to enhance forward displacement of the maxilla by sutural growth (Menéndez‐Díaz et al., 2018; Usman et al.,^2022^). This early intervention helps establish normal jaw relations by relieving impeded maxillary growth and producing a favorable periodontal status by eliminating traumatic occlusion (Graber & Vanarsdall, 2017; Ngan, 2005).

Recently, maxillary protraction using skeletal anchorage with a facemask or intraoral appliance has been introduced (De Clerck et al., 2012; Lee et al., 2022; Rutili et al., 2022). This is mostly performed in patients with permanent dentition, aged over 11 years. It is known to minimize dental effects (Seiryu et al., 2020; Wang et al., 2022), but could be disagreeable to parents of a younger age in early mixed dentition due to its invasive procedure.

**Figure 1:**
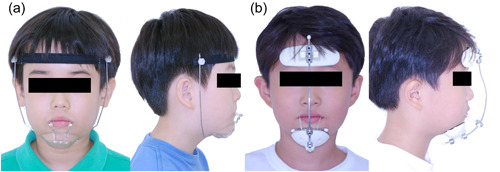
(a) Protraction facemask with forehead strap (PFFS). (b) Petit type facemask (PTF).

Various maxillary protraction headgear such as Delaire, forehead strap, and Petit‐type facemasks are currently available. The Delaire face mask (also called reverse headgear) consists of a forehead pad and chin cup connected by a square‐shaped bilateral framework with a connecting wire for elastic attachment. It offers good rigidity but is rather bulky and can cause difficulties with side‐sleeping and wearing eyeglasses (Proffit et al., 2012). Protraction facemasks with forehead straps (PFFS) are comprised of a forehead strap and chin cup. The forehead strap is connected by lock nuts on the bilateral framework wire upward and parallel to the mandible border from both sides of the chin cup (Figure 1a). Also, two traction wires extend directly from the chin cup, which can be bent and used to engage elastics (Hickam, 1991). It is esthetic, does not hinder vision, and has a unilateral adjustment. Petit type facemask (PTF) comprises a 0.25‐inch midline framework wire connecting the forehead and chin pads and a 0.075‐inch crossbar wire for elastic engagement (Figure 1b). It also uses the forehead and chin as support (Petit, 1983). It is simple and comfortable for those wearing eyeglasses but can easily dislodge and sometimes get cockeyed (Samuels et al., 1996).

The clinician should choose which type of facemask to use based on their strengths and weaknesses, assuming there is no difference in price. Moreover, the skeletodental changes after orthopedic treatment may be different because of distinct structures to transfer maxillary protraction force between PFFS and PTF, which have traction wires of the chin cup and crossbar of the midline framework, respectively. This might result in differential pressures on supporting areas resistant to protraction force. Unfortunately, as far as the author knows, few studies have compared treatment effects relative to various types of facemasks. Therefore, the purpose of this study is to compare the short‐term treatment changes with two structurally different types of facemasks, PFFS and PTF, especially in the early mixed dentition period. The null hypothesis of this study is that there would be no different changes between the two facemasks.

## 2 MATERIALS AND METHOD

### 2.1 Patient selection

This retrospective study was reviewed and approved by the institutional review board of Seoul National University Bundang Hospital (approval no. B‐1812‐513‐101). In addition, all guidelines of the Declaration of Helsinki were followed. The sample for this study was recruited from a population of patients diagnosed as having Class III malocclusion from February 2012 to March 2018. Consecutive patients were selected based on the following inclusion criteria: (1) anterior crossbite in early mixed dentition with eruption of permanent incisors and first molars, (2) diagnosed as having skeletal Class III malocclusion (ANB; A point‐nasion‐B point angle < 0°) and Angle Class III molar relationship, (3) treated with rapid maxillary expanders (RME) and Protraction facemasks with forehead straps (PFFS, Great Lakes dental technologies) or Petit type facemask (PTF, Kwang Myung DAICOM Inc) facemasks. In addition, patients were excluded if they had facial asymmetry, craniofacial syndromes, or previous orthodontic treatment.

The patients were instructed to turn the expansion screw once each day (0.25 mm expansion/day), and wear a maxillary protraction mask, assigned randomly PFFS or PTF, after expansion. The patients' compliance was checked every visit to ensure at least 12 hours a day. Maxillary protraction was accomplished with elastics from the hooks distal to the canines, 30° downward and forward from the occlusal plane, exerting about 400 g on each side.

### 2.2 Cephalometric measurements

**Figure 2:**
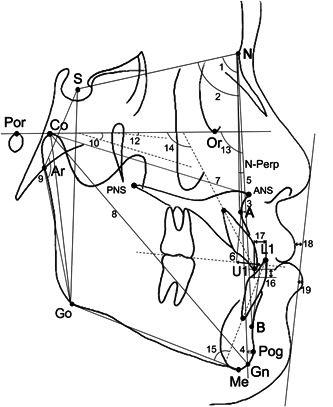
Cephalometric angular and linear measurements. 1. SNA, sella–nasion—A point angle; 2. SNB, sella–nasion–B point angle; 3. A point—N Perp, the distance between the A point and perpendicular line to the Frankfort horizontal plane (Por‐Or) from the Nasion (N); 4. Pog—N Perp, the distance between the Pogonion and the perpendicular line to the Frankfort horizontal plane (Por‐Or) from the Nasion (N), 5. ANB, A point–nasion–B point angle; 6. Wits appraisal, Distance between the projection of A and B points perpendicular to the occlusal plane; 7. Maxillary length, the distance between condylion (Co) and anterior nasal spine (ANS); 8. Mandibular length, the distance between Condylion (Co) and Gnathion (Gn); 9. Articular angle, sella (S)—articulare (Ar) ‐gonion (Go) angle; 10. FMA, the angle between Frankfort horizontal plane (Por‐Or) and mandibular plane (Go‐Me); 11. PFH/AFH, posterior facial height (S‐Go)/anterior facial height (N‐Me); 12. Palatal plane angle, the angle between Frankfort horizontal plane (Por‐Or) and palatal plane (PNS‐ANS); 13. Facial angle (Down's), the angle between Frankfort horizontal plane (Por‐Or) and N‐Pog; 14. U1 to FH, the angle formed by the intersection of the tooth axis of the upper incisor and Frankfort horizontal plane (Por‐Or); 15. IMPA, the angle between the long axis of the lower central incisor and mandibular plane (Go‐Me); 16. Incisor overbite; 17. Incisor overjet; 18. Upper lip EL, the distance between the upper lip to the esthetic line; 19. Lower lip EL, the distance between the lower lip to the esthetic line.

Lateral cephalograms were acquired before (T1) and after (T2) maxillary protraction. All cephalograms were traced and analyzed using V‐Ceph 6.0 (Osstem Implant Co, Ltd) by an experienced orthodontist (S. ‐H. K.) to evaluate the skeleto‐dental and soft tissue changes in each subject and to eliminate inter‐examiner errors. The cephalometric linear and angular measurements are shown in Figure 2. In addition, the same examiner, blinded as to which type of facemask was used relative to the treatment results, traced 15 randomly selected cephalograms after 2 weeks to evaluate intra‐examiner reliability. The intraclass correlation coefficient ranged from 0.912 to 0.991 for all cephalometric variables.

### 2.3 Statistical analysis

A Shapiro–Wilk test was used to determine the normality of the data distribution for all the variables. Depending on the normality of the data, a paired *t*‐test was used to evaluate the treatment changes (T2–T1). The pretreatment conditions (T1) and treatment changes of the cephalometric variables were compared using an independent *t*‐test between the two groups at a significant level of .05. Statistical analysis was performed using IBM SPSS Statistics for Windows, version 25.0 (IBM Corp).

The sample size calculation was performed with G* power 3.1.9.7 using .05 *α* level and a power of 80% (Faul et al., 2009), based on previous studies conducted with the same type of facemask at a similar age (Macdonald et al., 1999; Ngan et al., 1996). It showed that a minimum of 13 in each group were required.

## 3 RESULTS

A total of 40 subjects were selected and allocated into one of the two groups according to the type of facemask that had been used on them; the PFFS group (13 females, 7 males; age 7.70 ± 0.92) and the PTF group (13 females, 7 males; age 7.60 ± 1.05) (Figure 1a,b).

As shown in Table 1, there was no significant difference in mean age and dental or skeletal measurements between the PFFS and PTF groups before treatment (T1). In addition, the average treatment duration for both groups was 9.0 and 9.1 months, respectively.

**Table 1:** Comparison between protraction facemask with forehead strap (PFFS) and Petit type facemask (PTF) groups at pre‐treatment (T1)

	PFFS		PTF		*p* Value
	Mean	SD	Mean	SD	
Age before treatment (years)	7.7	0.92	7.6	1.05	NS
Treatment duration (months)	9	0.3	9.1	0.55	NS
SNA (°)	77.71	2.04	77.24	3.02	0.564
SNB (°)	78.82	2.50	78.26	2.63	0.492
A point—N Perp (mm)	−3.07	2.47	−3.22	3.71	0.888
Pog ‐ N Perp (mm)	−4.13	3.83	−4.10	6.64	0.985
ANB difference (°)	−1.11	1.77	−1.02	1.68	0.872
Wits appraisal (mm)	−8.82	2.45	−8.44	2.30	0.614
Maxillary length (mm)	78.26	3.51	78.44	3.92	0.876
Mandibular length (mm)	104.87	5.43	105.37	4.18	0.745
Articular angle (°)	146.29	4.33	144.26	6.30	0.243
FMA (°)	26.92	4.27	27.39	3.74	0.711
PFH/AFH (mm)	63.01	5.08	62.70	3.11	0.813
Palatal plane angle (°)	0.61	2.88	0.60	3.30	0.992
Facial angle (downs) (°)	87.70	2.13	87.79	3.58	0.924
U1 to FH (°)	110.52	6.10	112.99	5.73	0.288
IMPA (°)	86.36	6.13	86.94	6.40	0.809
Incisor overbite (mm)	2.34	1.60	1.72	2.00	0.375
Incisor overjet (mm)	−1.88	0.90	−1.40	1.40	0.279
Upper lip EL (mm)	−0.13	1.94	−0.42	2.47	0.687
Lower lip EL (mm)	2.22	2.19	2.48	2.75	0.74

*Note*: Refer to the legends of Figure 2 for the definition of each measurement.

Table 2 shows that the anteroposterior position of A point moved forward in the maxilla of both groups from T1 to T2, (*Δ*SNA, 1.37° and 1.41°; *Δ*A point to N perp, 1.15 and 1.46 mm; *Δ*maxillary length, 1.97 and 1.75 mm, respectively, all *p* < .001) and there was a decrease in the palatal plane angles (−1.01°, *p* < .001 and −0.87°, *p* < .05).

**Table 2:** Comparison of the differences in the skeleto‐dental changes in protraction facemask with forehead Strap (PFFS) and Petit type facemask (PTF) groups, and between the two groups from pretreatment (T1) to posttreatment (T2)

	PFFS			PTF			PFFS vs. PTF
T2–T1	Mean	SD	*p* Value	Mean	SD	*p* Value	*p* Value
*Δ*SNA (°)	1.37	0.98	0.000***	1.41	0.95	0.000***	0.887
*Δ*SNB (°)	−1.52	1.01	0.000***	−1.37	1.11	0.000***	0.674
*Δ*A point—N Perp (mm)	1.15	0.94	0.000***	1.46	1.35	0.000***	0.41
*Δ*Pog—N Perp (mm)	−2.41	1.65	0.000***	−1.91	2.29	0.001**	0.429
*Δ*ANB difference (°)	2.89	0.91	0.000***	2.79	1.13	0.000***	0.765
*Δ*Wits appraisal (mm)	3.65	2.09	0.000***	3.79	2.66	0.000***	0.848
*Δ*Maxillary length (mm)	1.97	1.18	0.000***	1.75	1.49	0.000***	0.603
*Δ*Mandibular length (mm)	1.27	1.36	0.001**	1.58	1.77	0.001**	0.534
*Δ*Articular angle (°)	1.43	1.95	0.004*	1.25	2.28	0.025*	0.788
*Δ*FMA (°)	1.67	0.87	0.000***	1.17	1.72	0.007**	0.256
*Δ*PFH/AFH (mm)	−0.55	0.78	0.005**	−0.54	1.39	0.097	0.981
*Δ*Palatal plane angle (°)	−1.01	0.89	0.000***	−0.87	1.66	0.030*	0.737
*Δ*Facial angle (downs) (°)	−1.18	0.87	0.000***	−1.00	1.37	0.004**	0.632
*Δ*U1 to FH (°)	3.79	4.56	0.005**	3.17	4.87	0.045*	0.735
*Δ*IMPA (°)	−3.19	3.26	0.001**	−3.54	2.22	0.000***	0.757
*Δ*Incisor overbite (mm)	−0.42	1.52	0.284	0.86	1.56	0.082	0.038****
*Δ*Incisor overjet (mm)	6.35	1.22	0.000***	5.95	1.70	0.000***	0.475
*Δ*Upper lip EL (mm)	1.99	1.19	0.000***	1.96	1.14	0.000***	0.943
*Δ*Lower lip EL (mm)	0.19	1.44	0.568	0.13	1.38	0.681	0.897

*Note*: Refer to the legends of Figure 2 for the definition of each measurement.

* *p* < .05

** *p* < .01

*** *p* < .001. Paired *t* test

**** *p* < .05. Independent *t* test.

In terms of the anteroposterior position of the mandible, the PFFS and PTF groups showed posterior repositioning of the mandible (*Δ*SNB, −1.52° and −1.37°, both *p* < .001; *Δ*Pog to N perp, −2.41 mm *p* < .001 and −1.91 mm, *p* < .01; *Δ*Facial angle, −1.18°, *p* < .001 and −1.00°, *p* < .01) and an increase in ANB (2.89° and 2.79°, both *p* < .001) and wits appraisal (3.65 and 3.79, both *p* < .001). The mandibular length increased in both groups (1.27 and 1.58 mm, both *p* < .01).

Regarding the changes in the vertical relationship, both groups presented opening rotation of the mandible (*Δ*FMA, 1.67°, *p* < .001 and 1.17°, *p* < .01; *Δ*articular angle, 1.43°, *p* < .01 and 1.25°, *p* < .05). The PFFS group exhibited a significant decrease in PFH/AFH (−0.55, *p* < .05).

Regarding the dental and soft tissue changes within each group, both groups had increases in overjet (6.35 and 5.95 mm, both *p* < .001), labioversion of the maxillary incisors (*Δ*U1 to FH, 3.79°, *p* < .01 and 3.17°, *p* < .05), and protrusion of the upper lips (*Δ*upper lip EL, 1.99 and 1.96 mm, both *p* < .001).

As shown in Table 2, there was a significant difference in overbites between the two groups (*p* < .05). PFFS group showed slightly decreased overbite (−0.42 mm), while the PTF group exhibited an increase in overbite (+0.86 mm). There was no significant difference in other measurements.

## 4 DISCUSSION

Facemask therapy has effectively treated growing skeletal Class III patients with maxillary deficiency, producing anterior movement of the maxilla, clockwise rotation of the mandible, and labial/lingual inclination of upper and lower anterior teeth (da Silva Filho et al., 1998; Macdonald et al., 1999). Until now, although various facemasks have been introduced and used on Class III patients, few studies have reported on the difference in treatment effects between them. Therefore, this study compared the treatment effects of the two types of facemasks; PFFS versus PTF.

There were no significant differences in mean age, treatment duration, and cephalometric variables between the PFFS and PTF groups at T1 (Table 1), indicating that the two groups were similarly matched in age and skeletal and dental Class III characteristics before facemask therapy.

In the present study, the PFFS and PTF groups showed the anterior movement of the maxilla and counterclockwise rotation of the palatal plane, but there were no intergroup differences (Table 2). These results were comparable to those of other previous studies with similar age groups. MacDonald et al. reported the treatment changes as SNA + 2.31°, Nperp to A + 1.91 mm, and SN‐PP −0.33° after 1 year of facemask treatment at the age of 7.4 (Macdonald et al., 1999). Another study also showed SNA + 1.09°, Nperp to A + 1.48 mm in Brazilian Class III patients after 8 months of treatment with Delaire‐type facemasks (da Silva Filho et al., 1998). Kapust et al. found SNA + 2.51°, Nperp to A + 1.96 mm, and SN‐PP ‐2.47° in 7–10 years compared to three age groups (Kapust et al., 1998). Nartallo‐Turley et al. also reported SNA + 2.35°, anterior movement of A point by +3.34 mm with the maxilla rotated counterclockwise, with PNS moving down more than ANS (−2.21 mm and −0.82, respectively) (Nartallo‐Turley & Turley, 1998). This rotated anterior palatal plane may have been caused by the extraoral force applied below the center of resistance of the maxillae (Braun, 2004). One study tried using a modified facemask to apply a higher force vector close to the center of resistance of the maxillae to minimize the maxillary rotation during the protraction (Keles et al., 2002).

Clockwise rotation of the mandible and an increase in the vertical dimensions were noted after facemask treatment in both groups (Table 2). These mandibular changes also corresponded with the already mentioned prior studies; MacDonald et al. (SNB –1.1°, Nperp to pog –1.86 mm, and FMA + 1.42°), Kapust et al. (Nperp to pog –2.59 mm and SN‐GoGn +1.42°), and Nartallo‐Turley et al. (SNB –1.32°, and significant downward movement at the menton –4.34 mm) (Kapust et al., 1998; Macdonald et al., 1999; Nartallo‐Turley & Turley, 1998). Mandibular length was also increased during the treatment period, that is, 9 months (1.27 mm in the PFFS group and 1.58 mm in the PTF group, *p* < .001). This mandibular growth fell into the range of mandibular growth reported by Mitani, 7 mm in 3 years (Mitani, 1981). No intergroup differences in skeletal variables were found between the groups. This suggests that the two types of facemasks induce advancement of the maxilla and posterior rotation of the mandible, that is, apparent treatment effects as they are opposite to natural growth (Nartallo‐Turley & Turley, 1998). However, the PFFS group exhibited slightly more posterior positioning with the vertical opening of the mandible (–2.41 mm in Pog to N perp; 1.67° in FMA; –1.18° in facial angle, all *p* < .001; –0.55 in PFH/AFH; 1.43° in articular angle, both *p* < .01) and more anterior rotation of the palatal plane (–1.01°, *p* < .001), compared to those of the PTF group. This suggests there might be a more significant increase in vertical dimension with PFFS than with PTF. It seems to be related to the design of the PFFS, in which the maxillary protraction force is exerted on the two traction hooks extended directly from the chin cup so that the mandible might be forced more backward in response to the anterior traction of the maxilla (Figure 1a). On the other hand, PTF has an adjustable crossbar wire on a framework connecting the chin cup and forehead pad, so the protraction force is applied at the crossbar level, away from the chin cup (Figure 1b).

Concerning dental and soft tissue changes, no intergroup differences were found except for overbite. Labioversion of the maxillary incisors, linguoversion of the mandibular incisors, and upper lip protrusion were comparable with results in previous reports (Franchi et al., 2004; Graber & Vanarsdall, 2017; Saadia & Torres, 2000). A significant difference was found in the overbite change after treatment between the two groups (*p* < .05); an 0.86 mm increase in the PTF group, during a –0.42 mm decrease in the PFFS group. This decrease in overbite in the PFFS group might be due to more anterior rotation of the maxilla and backward rotation of the mandible and labioversion of the maxillary incisors than was produced in the PTF group during the relatively short study.

To the best of our knowledge, this is the first report on short‐term treatment effects of PFFS and PTF facemasks that shows similar skeletal, dental and soft tissue changes with the two structurally different facemask types. They were both effective for maxillary protraction, but PFFS showed a significant decrease in overbite resulting from more counterclockwise maxillary rotation and clockwise mandibular rotation than with PTF. Considering that facemask does not modify the vertical growth pattern of each patient (Menéndez‐Díaz et al., 2018; Salazar et al., 2019), these results could be crucial to treating patients with hyperdivergent growth pattern or open bite. It seems that PTF can be considered to minimize vertical changes. This study has limitations such as the heterogeneous gender, small sample size, possible selection bias, and short‐term evaluation. Further studies could help compare treatment effects with other types of facemasks using a larger sample at different age groups and long‐term follow‐up skeleton‐dental and gingival changes.

## 5 CONCLUSIONS

This study failed to reject the null hypothesis. Both PFFS and PTF showed no significant differences in most skeletal and dental changes, except for overbite. These findings might be helpful for clinicians in selecting the types of facemasks for growing Class III malocclusion patients.

## AUTHOR CONTRIBUTIONS

**Nam‐Ki Lee**: Conceptualization; project administration; supervision; editing. **So‐Hyun Kim**: Data collection; data analysis. **Dong‐Whan Son**: Data collection; data analysis. **Jae Hyun Park**: Revision; editing. **Tae‐Hyun Choi**: Project administration; supervision; validation; writing; editing; revision. All authors have read and agreed to the published version of the manuscript.

## CONFLICT OF INTEREST

The authors declare no conflict of interest.

## Data Availability

The data sets generated and/or analyzed during the current study are not publicly available due to ethical requirements by the ethics committee.
